# Not so pseudo: the evolutionary history of protein phosphatase 1 regulatory subunit 2 and related pseudogenes

**DOI:** 10.1186/1471-2148-13-242

**Published:** 2013-11-06

**Authors:** Luís Korrodi-Gregório, Joana Abrantes, Thorsten Muller, José Melo-Ferreira, Katrin Marcus, Odete AB da Cruz e Silva, Margarida Fardilha, Pedro J Esteves

**Affiliations:** 1 Signal Transduction Laboratory, Centre for Cell Biology, Biology and Health Science Department, University of Aveiro, Aveiro, Portugal; 2 CIBIO-UP, Centro de Investigação em Biodiversidade e Recursos Genéticos, Universidade do Porto, InBIO, Laboratório Associado, Campus Agrário de Vairão, Vairão, Portugal; 3 INSERM, U892, Université de Nantes, Nantes, France; 4 Functional Proteomics, Medizinisches Proteom-Center, Ruhr-University Bochum, Bochum, Germany; 5 Neuroscience Laboratory, Centre for Cell Biology, Biology Department and Health Science Department, University of Aveiro, Aveiro, Portugal; 6 CESPU, Instituto de Investigação e Formação Avançada em Ciências e Tecnologias da Saúde, Gandra, Portugal

**Keywords:** PPP1, PPP1R2, Retroposons, Pseudogenization, Evolution, Vertebrates

## Abstract

**Background:**

Pseudogenes are traditionally considered “dead” genes, therefore lacking biological functions. This view has however been challenged during the last decade. This is the case of the Protein phosphatase 1 regulatory subunit 2 (PPP1R2) or inhibitor-2 gene family, for which several incomplete copies exist scattered throughout the genome.

**Results:**

In this study, the pseudogenization process of PPP1R2 was analyzed. Ten PPP1R2-related pseudogenes (PPP1R2P1-P10), highly similar to PPP1R2, were retrieved from the human genome assembly present in the databases. The phylogenetic analysis of mammalian PPP1R2 and related pseudogenes suggested that PPP1R2P7 and PPP1R2P9 retroposons appeared before the great mammalian radiation, while the remaining pseudogenes are primate-specific and retroposed at different times during Primate evolution. Although considered inactive, four of these pseudogenes seem to be transcribed and possibly possess biological functions. Given the role of PPP1R2 in sperm motility, the presence of these proteins was assessed in human sperm, and two PPP1R2-related proteins were detected, PPP1R2P3 and PPP1R2P9. Signatures of negative and positive selection were also detected in PPP1R2P9, further suggesting a role as a functional protein.

**Conclusions:**

The results show that contrary to initial observations PPP1R2-related pseudogenes are not simple bystanders of the evolutionary process but may rather be at the origin of genes with novel functions.

## Background

In the past, pseudogenes were generally regarded as functionally inert, due to the presence of several disabling features that prevent their expression (e.g. premature stop codons, frameshift mutations, no promoter regions, etc.), and therefore their evolution has been considered to be neutral [1]. However, this view has been challenged by new evidences, which demonstrate that certain pseudogenes are functionally active [1,2]. The GENCODE, a sub-project of the ENCODE (ENCyclopedia Of DNA Elements from the National Human Genome Research Institute, NHGRI), has estimated the number of pseudogenes in the human genome to be near 14,000 [3]. From these, ~6% were identified has potentially transcribed by computational models and almost half of them validated by RT-PCR-Seq techniques [3]. Indeed, pseudogenes can be functional at the DNA, RNA or protein levels and have a function related or independent of the parental gene [4]. At the DNA level, pseudogenes can regulate other genes by pseudogene insertion in the non-coding or coding region of the target gene and regulate the parental counterpart gene by gene conversion, homologous recombination and through regulatory sequences [4]. Concerning the RNA level, pseudogene RNAs can compete with the parental mRNA for miRNAs, RNA binding proteins and/or translational machinery binding, as well as, functioning as siRNAs and thereby inhibiting the parental gene expression. Pseudogenes can also function in unrelated genes as long non-coding RNAs, by encoding miRNA precursors or even compete for miRNAs [4]. At the protein level, pseudogenic proteins can have the same activity of the parental protein but function in different tissues, subcellular localization and/or pathophysiological conditions [5-11]. Pseudogenic proteins with altered functions might also affect the activity of the parental ones [12]. If a pseudogene mRNA is translated to a functional pseudogenic protein, this gene is often called a retrogene [13]. Pseudogenes can also produce truncated proteins that can function as antigenic peptides in the surface of the cells to stimulate the immune system against the malignant cells [4]. Pseudogenes have already been associated with several pathological conditions such as cancer [4], diabetes [14] and neurodegenerative diseases [15].

One promising model to understand the functional relevance of pseudogenization is the protein phosphatase 1 regulatory subunit 2 (PPP1R2). This protein, also known as inhibitor-2 (I2), was one of the first regulatory subunits identified as an inhibitor and binding partner of the Ser/Thr phosphoprotein phosphatase 1 (PPP1). PPP1R2 forms a stable complex with PPP1 catalytic subunit (PPP1C) blocking the active site and inhibiting it potently, being the reactivation triggered by phosphorylation [16-21]. The PPP1C/PPP1R2 complex has been implied in several processes such as cardiac function [22-24], mitosis and meiosis [25-30], tubulin acetylation and neuronal cell survival [31]. Also, it has been previously shown that a PPP1CC2/PPP1R2-like complex is important in the acquisition of sperm motility [32,33].

The PPP1R2 gene is conserved throughout all eukaryotes, from yeast to humans, with homologues found even in plants [34,35]. In the human genome, as observed for other ancient PPP1 inhibitors such as PPP1R8 (NIPP1) and PPP1R11 (I3), several sequences have been identified that are highly similar to PPP1R2 [34]. For PPP1R2, nine loci were found that present hallmark features of processed pseudogenes. These related sequences were collectively named PPP1R2 pseudogenes and were numbered from 1 to 9 (PPP1R2P1-P9) [34]. These pseudogenes are found scattered in the genome due to random retrotransposition phenomena that consist on the reverse transcription of cellular RNAs and random insertion into the nuclear genome [36,37]. Past studies identified four PPP1R2 pseudogenes at the messenger RNA level using high throughput techniques. PPP1R2P1 and PPP1R2P2 were discovered in human [38,39], PPP1R2P3 in human and crab-eating macaque (*Macaca fascicularis*) and PPP1R2P9 (also called I4) was found in human and mouse (*Mus musculus*) [40-43].

In this work we performed an exhaustive search for PPP1R2 pseudogenes in publicly available mammalian genome databases in order to infer their evolutionary history. In the collected pseudogenes, an assay for detection of the proteins was conducted. Our results show that evolution and pseudogenization of PPP1R2 gene may be correlated with the formation of new genes and the gain of new specific functions.

## Results and discussion

A total of 119 sequences were retrieved from the NCBI and Ensembl databases by blasting against the human PPP1R2 mRNA sequence. Ten pseudogenes were obtained from human sequences, increasing by one the previous number reported in the literature [34]. All pseudogenes obtained are intronless and with a truncated 5’UTR meaning that are processed pseudogenes. The parental human PPP1R2 CDS (618 bp) covers 17% of the entire mRNA (3475 bp); even the pseudogenes with the lowest coverage contain the parental CDS, with the exception of PPP1R2P7 that only comprises part of the 3’UTR.

### Phylogenetic analysis

In order to increase the reliability of the alignment for the phylogenetic reconstruction, we selected sequences with >85% coverage and >60% similarity with the human PPP1R2 CDS. By doing this, 81 sequences were included in the tree that represented all the pseudogenes with the exception of PPP1R2P7 (Additional file 1: Table S1). The unused sequences encompassed pseudogenic fragments and sequences where identity with PPP1R2 was detected mostly outside the CDS (e.g. PPP1R2P7) or presented truncated CDS (e.g. some PPP1R2P8 and PPP1R2P9).

**Figure 1:**
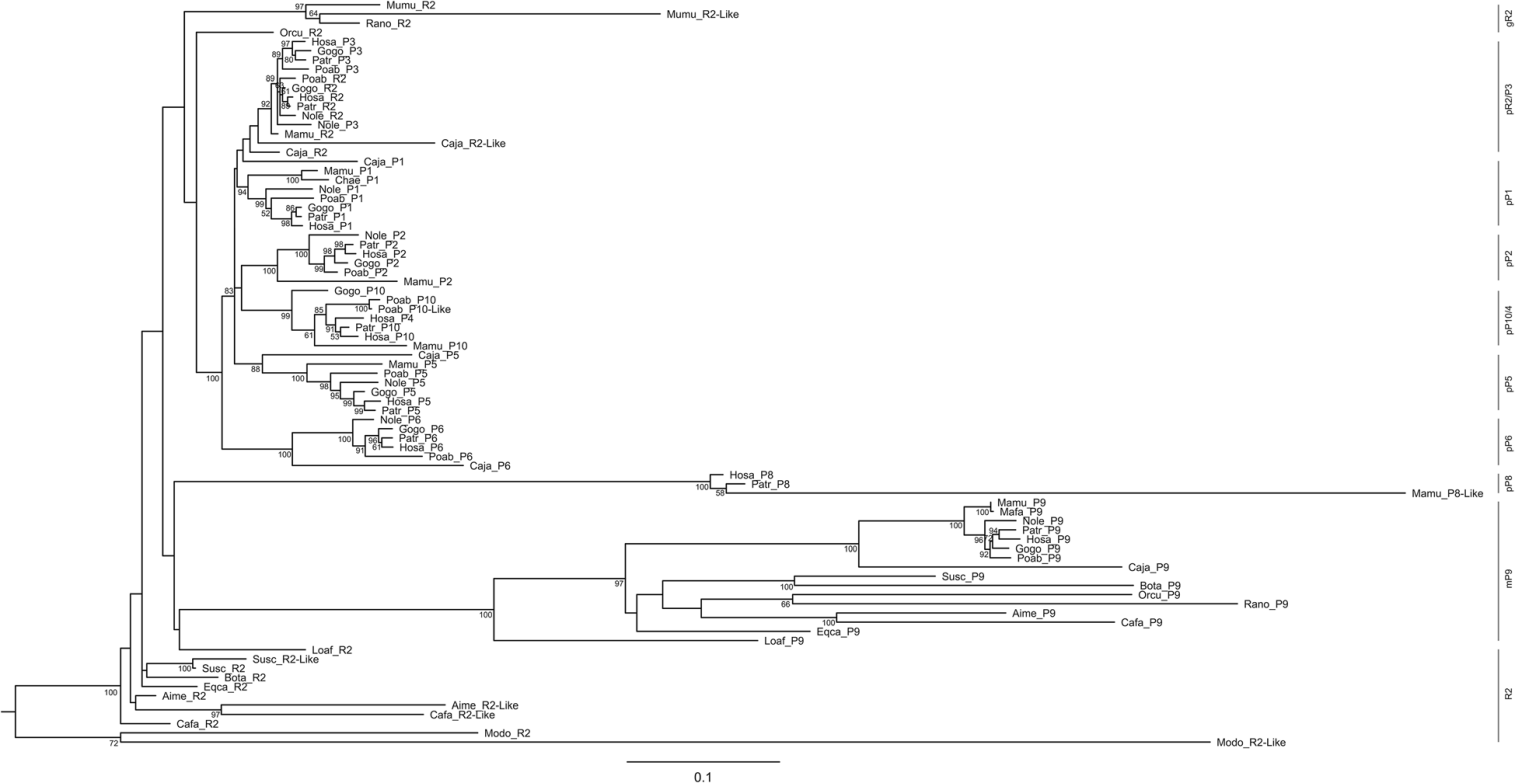
**Evolutionary tree of PPP1R2 and related pseudogenes.** The evolutionary history was inferred using the software GARLI. Best ML tree found in 1000000 generations is shown. Bootstrap values from 1000 replicates appear next to the nodes with values below 50% not shown. Group clusters are presented on the right, R2: PPP1R2 group; P1-P9: PPP1R2P1-P9 group. Low case letters before groups, p: primates; m: mammals; g: glires. Each sequence included in the tree is denoted by the first two letters of the genera followed by the first two letters of the species description and by the name of the gene same way as for the groups, Aime: *Ailuropoda melanoleuca*; Bota: *Bos taurus*; Cafa: *Canis familiaris*; Caja: *Callithrix jacchus*; Chae: *Chlorocebus aethiops*; Eqca: *Equus caballus*; Gogo: *Gorilla gorilla*; Hosa: *Homo sapiens*; Loaf: *Loxodonta africana*; Mado: *Monodelphis domestica*; Mamu: *Macaca mulatta*; Mumu: *Mus musculus*; Nole: *Nomascus leucogenys*; Orcu: *Oryctolagus cuniculus*; Patr: *Pan troglodytes*; Poab: *Pongo abelii*; Rano: *Rattus norvegicus*; Susc: *Sus scrofa*.

From the ML tree, four major clusters can be distinguished, generally supported by high bootstrap values (Figure 1). One of the clusters includes most mammalian PPP1R2 sequences, the exceptions being Primates PPP1R2, Glires PPP1R2, PPP1R2-like sequences (rabbit, *Oryctolagus cuniculus*, Orcu; rat, *Rattus norvegicus*, Rano; and mouse, *Mus musculus*, Mumu), and the elephant PPP1R2 (*Loxodonta africana*, Loaf). The other cluster comprises PPP1R2P8 and PPP1R2P8-like primate sequences. Mammalian PPP1R2P9 sequences compose a third cluster and a fourth cluster includes all PPP1R2 and related pseudogene sequences from Primates (PPP1R2P1/P2/P3/P4/P5/P6/P10). These sequences are clustered with the Glires PPP1R2 sequences. PPP1R2 is also present in the gray short-tailed opossum (*Monodelphis domestica*, Modo) which is consistent with the presence of PPP1R2 in eukaryotes, being indeed an ancient and well conserved gene [34].

**Figure 2:**
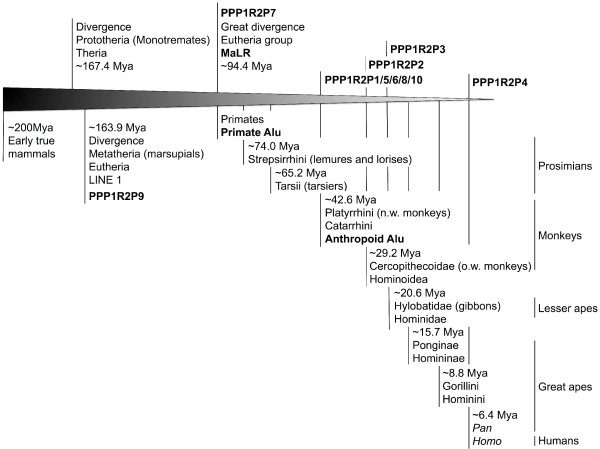
**Diagram of PPP1R2 pseudogenes evolution.** Time scale from the early mammals evolution till humans is shown with emphasis in the primate class. The time in million years ago (Mya) indicates the split between groups. Pseudogenes estimated emergence is shown, as well as, important retrotransposable elements.

**Figure 3:**
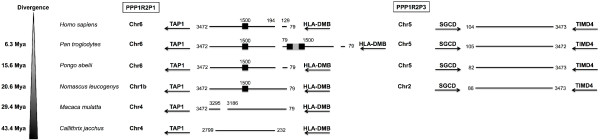
**Conserved linkage of PPP1R2P1 and PPP1R2P3.** PPP1R2P1 and PPP1R2P3 location in terms of chromosome and flanking genes is presented concerning each species where were found, showing the conserved linkage. Divergence time is shown on the left. The nucleotide number flanking the pseudogenes is related to the parental PPP1R2 message. Black boxes refer to the short interspersed elements (SINEs) Alu repeats that are primate-specific. Grey boxes refer to the long interspersed elements (LINEs), in this case a LINE1 element. Number above the boxes indicates the location where the repeat interrupted the sequence. In the case of chimpanzee PPP1R2P1, a duplicated pseudogene was originated and the repeats are located in the middle of both, and so, the numbers refer to the final of one pseudogene and the beginning of the other. Also, a deletion is shown (129 to 194) that is common to all pseudogenes with the exception of gibbon and marmoset and a deletion (3186 to 3295) also occurred in rhesus monkey. TAP1: transporter 1, ATP-binding cassette, sub-family B; HLA-DMB: major histocompatibility complex, class II, DM beta; SGCD: sarcoglycan delta; TIMD4: T-cell immunoglobulin and mucin domain containing 4.

**Figure 4:**
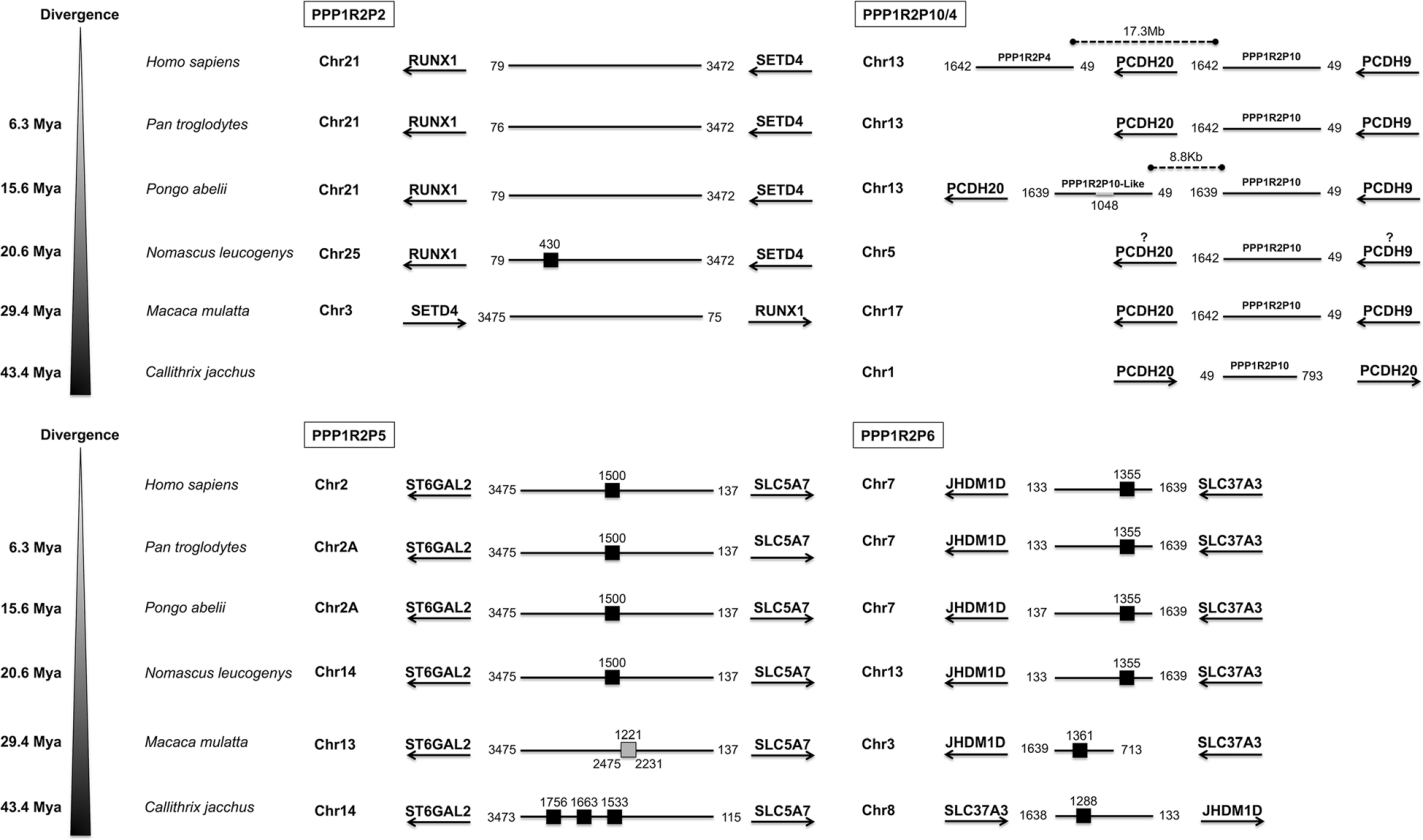
**Conserved linkage of PPP1R2P2, PPP1R2P10/4, PPP1R2P5 and PPP1R2P6.** PPP1R2P2, PPP1R2P10/4, PPP1R2P5 and PPP1R2P6 location in terms of chromosome and flanking genes is presented concerning each species where were found, to show the conserved linkage in these pseudogenes. Divergence time is shown on the left. The nucleotide number flanking the pseudogenes is related to the parental PPP1R2 message. Black boxes refer Alu repeats that are primate-specific. Number above the boxes indicates the location where the repeat interrupted the sequence. Grey box delimited with a black line in rhesus monkey PPP1R2P5 refer to a parental PPP1R2 insertion. Number on the top indicates where the insertion took place in the pseudogene, while numbers at the bottom show which region of the parental PPP1R2 was inserted. In orangutan an unknown sequence according to the current genome assembly was inserted in PPP1R10-like and is shown with a number on the bottom referring to the location. Gibbon PPP1R2P10 sequence was retrieved in a portion of the chromosome 5 not properly localized in the reference genomic sequence and so, even if the flanking genes were present in the same chromosome, the local could not be verified. The distances in dashed lines of the duplicated forms in human and orangutan are also indicated. RUNX1: Runt-related transcription factor 1; SETD4: SET domain containing 4; PCDH9/20: protocadherin 9/20; ST6GAL2: ST6 beta-galactosamide alpha-2,6-sialyltransferase 2; SLC5A7: solute carrier family 5 (choline transporter), member 7; JHDM1D: histone demethylase 1 homolog D; SLC37A3: solute carrier family 37 (glycerol-3-phosphate transporter), member 3.

**Figure 5:**
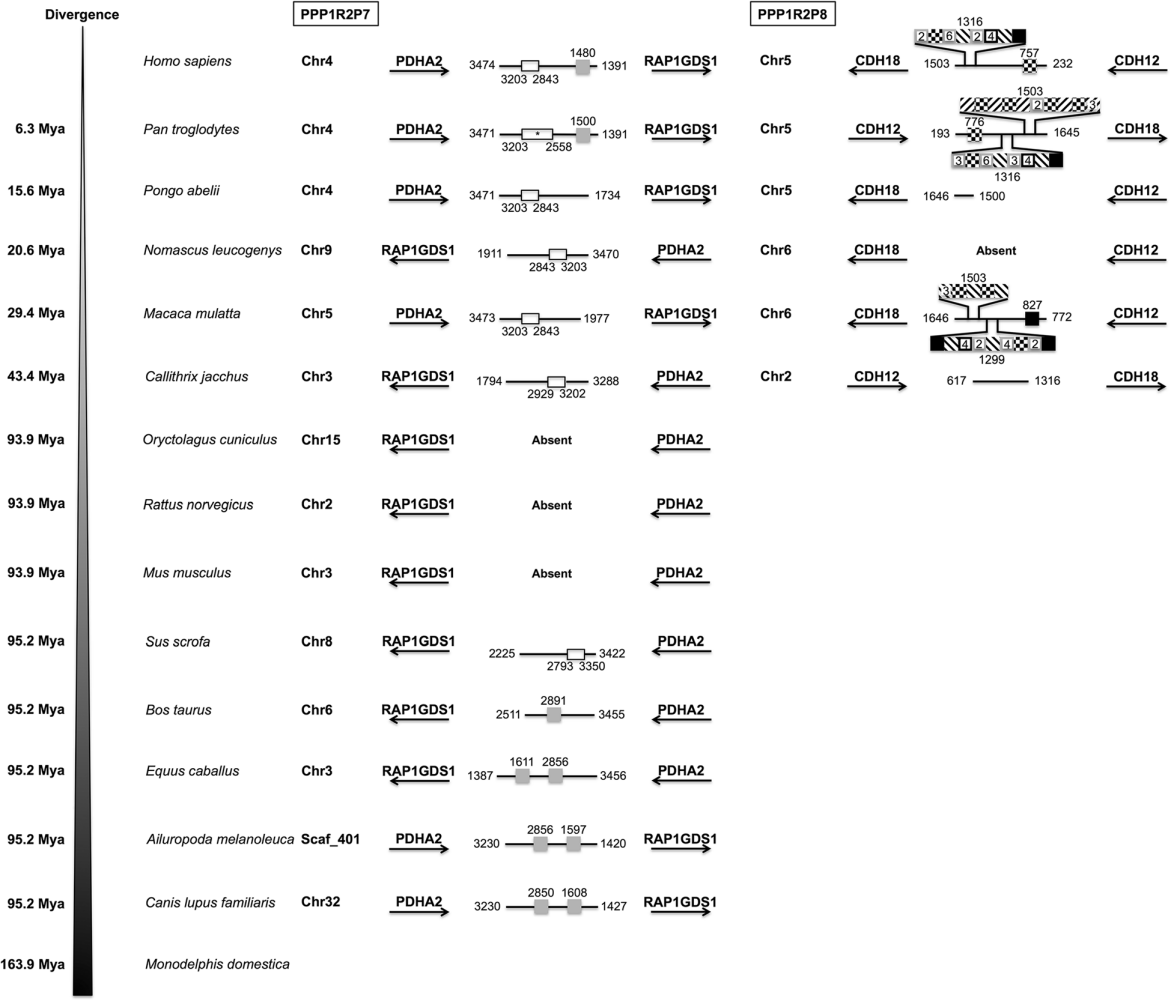
**Conserved linkage of PPP1R2P7 and PPP1R2P8.** PPP1R2P7 and PPP1R2P8 location in terms of chromosome and flanking genes is presented concerning each species where were found, showing the conserved linkage. Divergence time is shown on the left. The nucleotide number flanking the pseudogenes is related to the parental PPP1R2 message. Grey boxes refer to the long interspersed elements most LINE1 elements and one LINE2 element. Black boxes indicate SINEs most Alu repeats that are primate-specific but also others (e.g. MIR). Checkered box indicate long terminal repeat (LTR, from the ERV1, ERVL and MaLR families). Black diagonal traced white boxes indicate DNA-related repeats (hAT-Charlie and TcMar-Tigger families). Number above the boxes states the location where the repeat interrupted the sequence. Numbers inside the boxes indicate if there is more than one in line. White boxes delimited with a black line show a region that is absent and substituted by other unknown region. Numbers below the boxes show the region that is absent. * part of this sequence has unknown nucleotides and so the range (2558-3203 bp) might be similar to the other species (2843-3203 bp).

**Figure 6:**
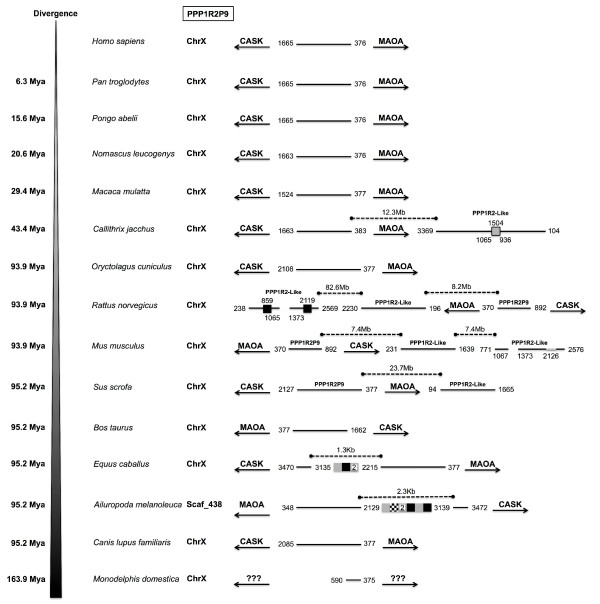
**Conserved linkage of PPP1R2P9.** PPP1R2P9 location in terms of chromosome and flanking genes is presented concerning each species where was found, showing the conserved linkage. Divergence time is shown on the left. The nucleotide number flanking the pseudogenes is related to the parental PPP1R2 message. Grey boxes refer to the LINE1 elements. Black boxes refer to SINEs B2 repeats that are rodent-specific and to the tRNAs present in the horse (*Equus caballus*) and in the giant panda sequences. Checkered box refers to long terminal repeat (LTR) in the giant panda sequence, which is an endogenous retroviral-related element (ERVL). Number above the boxes states the location where the repeat interrupted the sequence. Numbers inside the boxes indicate if there is more than one in line. Grey box delimited with a black line in marmoset PPP1R2P9-like refer to a parental PPP1R2 insertion. Number on the top refers where the insertion took place in the pseudogene, while numbers at the bottom show which region of the parental PPP1R2 was inserted. In mouse an unknown sequence according to the current genome assembly was inserted in PPP1R2P9-like and is shown with a number referring to its location. Also, a deletion is shown in mouse (1067 to 1373) and in rat (1065 to 1373) PPP1R2P9-like pseudogenes. The distances in dashed lines of the other retroposed forms in marmoset, mouse, rat and pig are also indicated. CASK: calcium/calmodulin-dependent serine protein kinase; MAOA: monoamine oxidase A.

Two major retroposition events can be inferred, the retroposition that originated PPP1R2P9 and the retroposition that gave origin to PPP1R2P1/P2/P3/P4/P5/P6/P10 (Figure 2). Retroposition of PPP1R2P9 occurred before the split of Eutheria (placental mammals) from Metatheria (marsupial mammals) at ~163.9-167.4 millions of years ago (Mya), as suggested by the presence of this pseudogene in the marsupial gray short-tailed opossum and in all other mammals, making PPP1R2P9 the most ancient pseudogene still present in humans (Figure 2). In the X chromosome we found, close to PPP1R2P9, more PPP1R2-like copies that seem to have arisen by PPP1R2 gene duplication: marmoset (one copy), rat (two copies), mouse (two copies) and pig (one copy). The phylogenetic analysis shows that these copies are related to parental PPP1R2 gene suggesting that this gene has been retroposed to the X chromosome more than once independently and at different time points in these species. We checked for gene conversion events and we did not find any evidence for it. In the phylogenetic tree the PPP1R2P9 genes are clearly apart of these PPP1R2-like that are clustered in the PPP1R2 gene group. PPP1R2P7 is also non-primate specific. Indeed, PPP1R2P7 was present in all mammalian orders included in this study, with the exception of Glires and opossum, suggesting that it was originated ~94.4-163.9 Mya (Figure 2). Retroposition of PPP1R2P1/P2/P3/P4/P5/P6/P10 is more recent and occurred in the ancestor of Primates and during Primates’ evolution since these pseudogenes occur only in primate species (Figure 2). Other retroposition events of PPP1R2 gene have also occurred in some mammals (pig, *Sus scrofa*, Susc; dog, *Canis lupus familiaris*, Cafa; giant panda, *Ailuropoda melanoleuca*, Aime; marmoset, *Callithrix jacchus*, Caja; and mouse; shown in the tree as R2-like) and appear to be species-specific events since these fragments are not widespread in mammals and both copies present in each species cluster together. Clustering of Glires PPP1R2 and R2-like pseudogenes along with PPP1R2P1/P2/P3/P5/P6/P10/P4 from Primates is consistent with the grouping of these species within the Euarchontoglires (or Supraprimates) superorder [44]. PPP1R2P1 was originated before the separation of New World monkeys (Platyrrhini) and Catarrhini that occurred 43.4-65.2 Mya (Figure 2). A 70 bp deletion seems to have occurred in Hominidae after the divergence from Hylobatidae, ~20.6 Mya. Also, an Alu repeat was inserted after the radiation of the Hominoidea, ~29.4 Mya, in the middle of the sequence disrupting it, but without affecting the open reading frame (ORF) (Figure 3). Interestingly, in chimpanzee, PPP1R2P1 suffered a recent duplication event that gave rise to a second locus separated by two Alu repeats flanking a LINE1 (long interspersed nuclear element, family L1) element (Figure 3, not included in the ML tree). Concerning PPP1R2P3, we found that it clusters along with Primates’ PPP1R2 suggesting that this is the most recent retroposed pseudogene originated after the separation of Hominoidea from Cercopithecoidea (old world monkeys), ~0.6-29.2 Mya, since no copy was found in rhesus monkey and marmoset (Figures 2 and 3). Clustering of PPP1R2P2 and PPP1R2P10/P4 might indicate that these pseudogenes arose by duplication. Our analysis shows that PPP1R2P10 is the ancestral, being originated before the division of Platyrrhini and Catarrhini (42.6-65.2 Mya), while PPP1R2P4 is a duplication that occurred only in humans, being therefore a duplicated pseudogene (Figures 1, 2 and 4). Also, in orangutan, a duplication occurred very close to PPP1R2P10 (~8.8 kb) that is not related with human PPP1R2P4, and was hence here named PPP1R2P10-like (Figures 1 and 4). The other pseudogenes (PPP1R2P5, PPP1R2P6 and PPP1R2P8) were originated at the same time as PPP1R2P10 (Figures 2, 4 and 5). PPP1R2P2 was originated in Catarrhini after its separation from the Platyrrhini ~29.2-42.6 Mya (Figures 2 and 4). The PPP1R2P7 (Glires) and PPP1R2P8 (gibbon) sequences were not retrieved from the databases, which suggest the later deletion of these pseudogenes (Figure 5). The fact that some genome annotations are early assemblies, might explain the missing of these and other sequences. However, the good quality of Glires (*Mus*, *Rattus* and *Oryctolagus*) genome assemblies reinforces the absence of PPP1R2P7 sequence and suggests that it occurred in the common ancestor. The absence of gibbon PPP1R2P8 sequence could also be explained by the several insertions present, similar to what happens in other species, virtually dismantling it and making the retrieval impossible (Figure 5). Moreover, the conserved linkage confirms the results of the phylogenetic analysis, being all pseudogenes flanked by the same respective genes in all species analyzed (Figures 3, 4, 5, 6).

### Evidences for functionality of PPP1R2-related pseudogenes

Features such as the existence of transcriptional related data, presence of regulatory elements, mRNA stability (e.g. UTRs, polyA signals), translation initiator sequence and complete ORFs (no truncations or disabling mutations) are indicators of the putative functionality of genes. A search for such features was conducted in order to verify the potential functionality of the PPP1R2 pseudogenes.

#### *PPP1R2P1*

The Gene Expression Omnibus (GEO, NCBI) and Gene Expression Atlas (GXA, Ensembl) public repositories contain expression data for PPP1R2P1. The presence of promoters, enhancers and other regulatory elements could be an explanation for PPP1R2P1 transcriptional related data (174 GEO and 2 GXA), although basal transcription should not be set aside. Concerning the mRNA stability, only part of the 5’UTR (238 bp), due to the low processivity of the reverse transcriptase, and part of the 3’UTR (506 bp) are present. Therefore, the stability might be compromised although a polyA signal (ATTAAA) is present near the 3’UTR terminus (position 1361, Figure 3). Regarding the translation, the Kozak sequence, important for translation initiation, is present in the parental gene and is conserved in PPP1R2P1. Altogether, these results suggest that at least in humans, PPP1R2P1 is expressed and might be functionally relevant. Although we cannot set aside the low quality of some of the assembled genomes, in other primates the ORF of PPP1R2P1 has frameshift disruptions that introduce premature stop codons, indicating that in these species might not produce a putative functional protein, or if so the protein might be truncated.

#### *PPP1R2P3*

The sequence of PPP1R2P3 is complete, without any frameshifts or element repeats disruptions (Figure 3). The sequence was truncated at the 5’UTR, as expected due to the low processivity of the reverse transcriptase, and in the 3’UTR it lost two of the four polyA signals that may lead to a short ~1500-1600 nt message. We have previously found, by a yeast two hybrid screening of human testis cDNA, using as bait PPP1CC1, one clone assigned to PPP1R2P3 [43,45]. A search for PPP1R2P3 ESTs in databases revealed that this is one of the most represented PPP1R2 pseudogenes (72 GEO, 313 GXA), being highly detected in testis (14 ESTs in Unigene). Together with our previous data, this strongly suggests that this pseudogene is transcribed. Two independent reports using mass spectrometry have also assigned peptides to PPP1R2P3 [46,47]. However, these peptides share the sequence with both parental gene and PPP1R2P3, being most probably misassigned. Nonetheless, we have shown recently by mass spectrometry the presence of PPP1R2P3 in human sperm samples [48].

#### *PPP1R2P9*

The PPP1R2P9 sequences retrieved have not been disrupted, at least in primates (Figure 6). However, the 5’UTR of the parental gene is absent and the 3’UTR is truncated (671 bp in humans). At the 3’UTR there is a single polyA signal at nucleotide position 1088, according to the human sequence, which suggests that a shorter message is produced. Sequence repeats, deletions, unknown and known sequence insertions were only found in the PPP1R2-like sequences (Figure 6). The only exceptions are in mouse and rat where the 3’UTR was deleted in the parental PPP1R2P9 (Figure 6).

This pseudogene is the one with more transcriptional related data (1086 GEO, 128 GXA) and has many ESTs in testis (9 ESTs in Unigene) like PPP1R2P3. PPP1R2P9 was originally found in cDNA libraries of human germ cell tumors, binding to PPP1C directly and in heat stable extracts inhibits this phosphatase potently with an IC50 of 0.2nM [40]. Also, we have recently identified PPP1R2P9 as an interacting partner of PPP1CA by yeast-two hybrid in human brain [49]. This suggests that silent regulatory areas are present in the region were PPP1R2P9 was retroposed and that during the evolution PPP1R2P9 might have retained or gained the capacity to be transcribed. In spite of this, there is no data suggesting the translation of PPP1R2P9. Considering the ORF, all species show a continuous ORF with no or small truncations at the C-terminus (e.g. in mouse and rat), with the exception of pig where no protein translation was obtained from the ORF.

### Evidences of non-coding nature of PPP1R2-related pseudogenes

Considering the other pseudogenes sequences, many insertions in PPP1R2P8 lead to a completely disrupted ORF and missense mutations in PPP1R2P4/P10 and PPP1R2P6 lead to premature stop codons (Figures 4 and 5). Also, since these pseudogenes showed low coverage to the parental gene, most of the 3’UTR is missing and so, regulatory elements such as polyadenylation signals that are important for the transcript cleavage and stability are absent. This indicates that no transcription or translation should be expected from these pseudogenes, which corroborates with the fact that no expression was found in ESTs and high-throughput databases with the exception of PPP1R2P4. Considering the pseudogenes with the highest coverage in relation to the parental gene, PPP1R2P2 and PPP1R2P5, no ORF disruptions were found but many missense mutations were found in all species analyzed that lead to premature stop codons (Figure 4). All four polyadenylation signals present in the parental PPP1R2 mRNA are conserved in PPP1R2P2. Although protein expression is unlikely, PPP1R2P2 message was found by qPCR in human testis but not in peripheral blood leukocytes [39]. Also, two experiments from ArrayExpress, report the up/down regulation of this pseudogene in prostate adenocarcinoma and in a prostate transcriptomic study performed in a Caucasian population [50]. These results might be artifacts or could be due to other PPP1R2 pseudogenes/parental gene since this pseudogene is located in chromosome 21 that has low density (~232 genes, only surpassed by the Y chromosome with 130 genes), and as expected, the processed pseudogene density is also low, 34 [51], making the transcription highly unlikely.

### Detection of PPP1R2-related proteins

**Figure 7:**
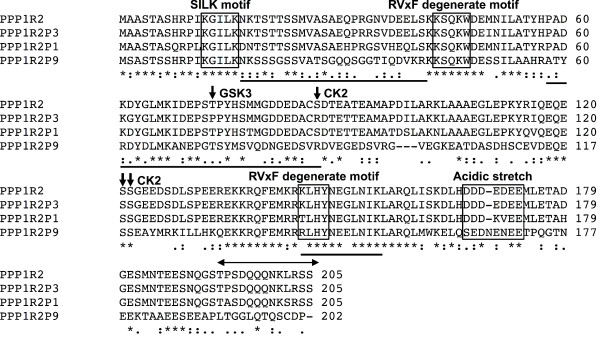
**Alignment of PPP1R2-related proteins reveals high conservation.** An alignment was performed using the protein sequences of PPP1R2P1, PPP1R2P3, PPP1R2P9 and PPP1R2. Black arrows indicate the important phosphorylation sites in PPP1R2 and the respective known kinase. Black boxes encage each PPP1 binding motif known for PPP1R2 and the acidic stretch. Black bars at the bottom of each row of alignment show the region covered by the peptides obtained. Two-headed arrow indicates the peptide for which the antibody used for immunoprecipitation was raised. * represent high conservation, : and . represent low conservation in which the substituted residue has respectively more or less similar properties.

PPP1R2 forms a stable and high affinity complex with PPP1C by blocking the active site. The reactivation of the complex is triggered by phosphorylation at Thr72 of PPP1R2 through several kinases, including glycogen synthase 3 (GSK3) [52-54]. PPP1R2 is also phosphorylated at the residue Ser86 by casein kinase 2 (CK2) that accelerates the subsequent phosphorylation at Thr72 by GSK3 [16]. The comparison of human PPP1R2P1, PPP1R2P3 and PPP1R2P9 with PPP1R2 amino acid sequences (Figure 7) shows that PPP1R2P9 is the most divergent (41%) and PPP1R2P3 the most similar (95%). Regarding the PPP1 binding motifs, SILK and KSQKW, they are conserved in all PPP1R2-related proteins, and KLHY is conserved in PPP1R2P3 but a substitution of the first residue to Thr or Arg is observed for PPP1R2P1 or PPP1R2P9, respectively [55]. The C-terminal acidic stretch (DDDEDEE) required for GSK3 phosphorylation [55,56] is maintained in PPP1R2P3 although the GSK3 phosphorylation site Thr73 is substituted to Pro. The other two pseudogenes maintain the GSK3 phosphorylation site but the acidic stretch has several changes particularly in PPP1R2P9 (Figure 7). Finally, the CK2 phosphorylation site Ser87 is conserved in PPP1R2P1 but is substituted by an Arg in PPP1R2P3 and PPP1R2P9. Overall, the analysis shows that these PPP1R2-related proteins should maintain the ability to bind to PPP1C, as was already demonstrated for PPP1R2P3 and PPP1R2P9 [40,48], and the ability to regulate the holoenzyme activity by GSK3 phosphorylation is compromised in PPP1R2P3 [48], and may also be but in a lesser extent in PPP1R2P9, due to the change Ser87 to Arg.

PPP1CC2 is a sperm-specific protein phosphatase involved in spermatogenesis and sperm motility [32,33,57]. Its inhibition *in vivo*, was associated with a PPP1R2-like activity since GSK3 was able to reverse the process [32]. Recently, a report identified the PPP1R2 protein in heat-stable extracts of bull testis and mouse testis and sperm where it may account for this PPP1R2-like activity [58]. It is well known that testis is one of the organs where most pseudogenes are expressed and their gene products were shown to have important roles in spermatogenesis and other germ cell related functions [52-54]. This might be due, in part, to the hyper-transcription state of the autosomal chromosomes in the meiotic and post-meiotic germ cells due to chromatin modifications [13,54,59]. A recent study done by GENCODE has revealed that 64% of all validated expressed pseudogenes are expressed in testis [3]. PPP1R2 is one of the PPP1C regulators with more related pseudogenes [34]. We have previously identified PPP1R2P3 message and protein, in testis [43,45,48]. We hypothesized that from the other pseudogenes, only PPP1R2P1 and PPP1R2P9 are capable of being also translated. In fact, the two pseudogenes, PPP1R2P3 and PPP1R2P9, were present in the mass spectrometry data obtained from a human sperm immunoprecipitation (Table 1).

**Table 1:** PPP1R2P9 presence in human sperm

***Protein name***	***Uniprot ID***	***MW *** **( *Da *)**	***pI***	***Protein size *** **( *aa *)**	***Coverage***	***Mascot score***
PPP1R2P9	IPP4_HUMAN	22,660	5.04	202	36.5%	623.41
***Peptide***		***Range *** **( *start *- *end *)**	***Number of spectra***	***m/z meas.***	***z***	***Mascot score***
*K.NKSSSGSSVATSGQQSGGTIQDVK.R*		*17–40*	*6*	*770.71*	*3*	*21.95*
*K.SSSGSSVATSGQQSGGTIQDVK.R*		*19–40*	*6*	*1,034.49*	*2*	*99.44*
*K.SSSGSSVATSGQQSGGTIQDVKR.K*		*19–41*	*4*	*1,112.54*	*2*	*38.09*
*R.LHYNEELNIK.L*		*143–152*	*2*	*424.89*	*3*	*31.40*
*K.ANEPGTSYMSVQDNGEDSVRDVEGEDSVR.G*		*68–96*	*2*	*1,053.45*	*3*	*63.05*
*R.RLHYNEELNIK.L*		*142–152*	*1*	*476.92*	*3*	*29.73*
*R.ATYRDYDLMK.A*		*58–67*	*1*	*431.20*	*3*	*28.17*
*K.ANEPGTSYMSVQDNGEDSVR.D*		*68–87*	*1*	*1,086.46*	*2*	*69.26*

Peptides were identified by Orbitrap Velos mass spectrometry, from human sperm heat stable extracts and immunoprecipitates using rabbit anti-PPP1R2 antibodies. aa, amino acids; pI, isoelectric point.

This analysis was based on the fact that the molecular weight of these PPP1R2-related proteins should be similar to the parental one (PPP1R2-23.0kDa), being therefore present in the same region where the band was extracted to mass spectrometry analysis. The antibody used to immunoprecipitate PPP1R2-related proteins was raised against a peptide containing amino acid residues 134–147 from the mouse PPP1R2 sequence (Figure 7). This antibody was used previously to detect PPP1R2 [48,58]. In the 14-residue region, PPP1R2P1 and PPP1R2P9 have two and three substitutions respectively, when comparing to PPP1R2 sequence. We predicted that using this antibody, we were also able to detect the other PPP1R2-related proteins. Mass spectrometry data identified 23 MSMS (tandem mass spectrometry) spectra corresponding to 8 different peptides matching unequivocally to PPP1R2P9 (Figure 7 and Table 1) and 3 MSMS spectra corresponding to one peptide matching unequivocally to PPP1R2P3 [48].

The sequence coverage obtained for PPP1R2P9 was 36.5% and the mascot score levels were 623.41 (in addition, spectra were manually evaluated). This is the first time that PPP1R2P9 protein is detected, being clearly recovered from human ejaculated sperm. Additionally, these results also indicate that native PPP1R2-related proteins are indeed heat stable and migrate at the same position as the parental PPP1R2. Lastly, no peptides were recovered for PPP1R2P1 using this method, which might suggest the absence of the protein, at least from the sperm cells.

### Signatures of selection

Pseudogenes have been regarded as being derived from functional-encoding genomic DNA sequences that have accumulated disabling mutations (frameshifts and premature stop codons) that make them non-coding protein genes. This lack of function predicts that pseudogenes are not under selective pressures and thus evolve neutrally (reviewed in [1]). Nevertheless, this view keeps being challenged by the accumulation of examples of transcribed pseudogenes with several acknowledged functions (e.g. regulation of the expression of paralogous genes through the generation of small-interfering RNAs) [1]. Signatures of selection, in addition to sequence conservation, have been considered as obvious indicators of the functional importance of pseudogenes [60].

Here, by using six ML methods, signatures of both positive and negative selection were detected in the PPP1R2P9 pseudogene, as well as in the parental gene PPP1R2 (Additional file 2: Table S2). Signatures of negative selection were far more evident than those of positive selection, for both genes. Four methods, REL, FEL, SLAC and FUBAR, showed sites negatively selected, with most being detected by more than one method. Signatures of positive selection were principally detected by FEL and MEME methods. The codons 92 and 120, for PPP1R2, and the codons 6, 208 and 211, for PPP1R2P9, were detected by at least two separate methods. No detection was obtained for the PAML method. It is known that sperm-expressed genes present in chromosome X tend to be positively selected when compared with X-linked non-sperm genes and with sperm-expressed autosomal genes [61,62]. This evolutionary pressure is due to their hemizigous expression in males that will favor advantageous mutations and remove any deleterious one. PPP1R2P9 is not evolving neutrally and may thus be expressed, further supporting a functional role for this pseudogene.

## Conclusions

Retropositions from the PPP1R2 gene are ancient, prior to the great radiation of the mammals, as supported by the presence of PPP1R2P9 and PPP1R2P7 in the different groups of mammals. All the other pseudogenes found in humans are primate-specific and were retroposed at different times during the evolution of this group. For instance, PPP1R2P3 exists only in the members of the Hominoidea family, whereas PPP1R2P8, the most distinct, is present in all groups and was retroposed ~42.6-65.2 Mya. This reveals that retropositions have occurred in waves and in a unique way similar to the Alu repeats explosion that occurred ~40-50 Mya, after the divergence of simian ancestors from the prosimians (lemurs and lorises). The recent pseudogene duplication in humans, PPP1R2P4, and in chimpanzee, PPP1R2P1, suggests that evolution of pseudogenes is still an active process.

As suggested by the presence of an uninterrupted ORF, ESTs and polyA signals, PPP1R2P9 (along with PPP1R2P1 and PPP1R2P3) appears to be transcribed. Moreover, the finding of positive and negative selection signatures suggests that it could be functionally relevant. Indeed, we confirmed that two PPP1R2-related proteins are translated in human sperm (PPP1R2P3 and PPP1R2P9), and are heat stable in their native form [48]. The importance of these PPP1R2-related proteins in physiological conditions, such as spermatogenesis and sperm physiology, should be assessed in future studies. Besides this, PPP1R2P1, PPP1R2P3 and PPP1R2P9 were found to be associated with pathological conditions [15,38,40,42,63]. Thus, assessing their ratios may be considered as a diagnostic tool in the future.

Furthermore, it has been shown that pseudogenes can regulate their parental counterparts at the RNA level either by siRNA or by competition for positive and negative stabilizing factors and miRNAs [64]. Although PPP1R2P2, PPP1R2P4 and PPP1R2P10 translation is very unlikely, their expression is documented and so, it is feasible these pseudogenes could regulate the parental PPP1R2 message levels and therefore its function.

These observations indicate that PPP1R2 pseudogenes have possible biological functions rather than acting as non-functional relics as initially believed. Their evolution process might be in part related with the formation of new genes and the gain of new specific functions. Therefore, their designation as pseudogenes should be reevaluated.

## Methods

### Sequences retrieval

The human PPP1R2 mRNA sequence (GenBank accession number NM_006241.4) was used to detect orthologs and pseudogene-related sequences by performing a BLAST search on GenBank, from National Center for Biotechnology Information (NCBI, http://BLAST.ncbi.nlm.nih.gov/) and Ensembl (http://www.ensembl.org/Multi/blastview) databases against all available mammalian reference genomic sequences. Only sequences with more than 60% of sequence similarity and with query coverage of more than 35% were recovered. Genomic sequences flanking the retrieved sequences were also manually inspected for missing parts, especially at the 3’UTR.

### Evolutionary tree reconstruction and divergence times

The retrieved sequences (Additional file 1: Table S1) were visually inspected and aligned using ClustalW implemented in BioEdit 7.0.9.0 [65]. For phylogenetic reconstruction, and to improve accuracy, only sequences encompassing >85% coverage of the human PPP1R2 CDS (nucleotide positions 377–994 of the mRNA sequence) and with >60% of sequence similarity were included in the alignment. In order to determine the phylogenetic relationships between the PPP1R2 gene and related pseudogenes, the best-fit model of nucleotide substitution was first assessed using the program jModelTest v0.1.1 [66] under the Akaike Information Criterion (AIC). A maximum likelihood (ML) phylogeny was inferred using the software GARLI v1.0 [67] by indicating the best nucleotide substitution model. No starting topology was defined and the program was set to run until no significant topology improvement (as defined by the default settings) was found after 1000000 generations. Five independent runs were performed to check the consistency of the estimates. The support of each node was assessed using 1000 bootstrap replicates. For each bootstrap replicate, the number of generations was set at 100000, above the generation where the last topological improvements were found for each of the five independent replicates. A 50% majority-rule consensus tree of the 1000 bootstrap replicates was created using PAUP* [68]. The support values at each node of the consensus tree were added to the best tree found by GARLI.

Divergence times from the other species in relation to *Homo sapiens* in millions of years ago (Mya) were obtained from TimeTree (http://www.timetree.org/) [69].

### Pseudogene classification and conserved linkage

Sequences obtained from the BLAST queries were analyzed in terms of presence of intronic regions, polyA traits (PolyApred, http://www.imtech.res.in/raghava/polyapred/), truncation of the 5’UTR and chromosomal location. Chromosomal locations were obtained from the GenBank database (Additional file 1: Table S1). Pseudogenes located in the same chromosome and nearby and/or with intronic regions were classified as duplicated pseudogenes. Pseudogenes that were located in different chromosomes and had polyA traits, truncation of the 5’UTR and no introns were classified as processed pseudogenes. Furthermore, genes flanking each human PPP1R2 pseudogene and conserved among mammals were selected. Conserved linkage, meaning conservation of synteny and also conservation of the gene order, was then searched for in order to provide insights regarding their orthology.

### Distance to closest and repeated regions

The distance of each pseudogene to the closest neighboring gene, not taking into account the presence of nearby pseudogenes, was calculated. Repeated sequences were detected by submitting each pseudogene sequence to the program RepeatMasker from Institute for Systems Biology, Seattle, Washington, USA (http://www.repeatmasker.org/).

### Signatures of natural selection

Coding sequences evolving neutrally present a ratio (ω) of non-synonymous (dN) over synonymous substitutions (dS) that do not significantly deviate from one. An excess of non-synonymous substitutions over synonymous substitutions (dN > dS) might indicate positive selection, suggesting that the replacement might be advantageous, while negative selection results from the scarcity of non-synonymous substitutions (dN < dS), indicating that a particular mutation most likely is deleterious and is being removed from the gene pool. Pseudogenes are considered to evolve neutrally (reviewed in [1]).

Maximum-likelihood codon-based tests were used to test for statistically significant signatures of selection in PPP1R2 and related-pseudogenes. Nevertheless, only PPP1R2P9 sequences were analyzed since at least 10 sequences are required to robustly detect signatures of selection [70]. Signatures of positive and negative selection were searched for in Datamonkey webserver (http://www.datamonkey.org) that uses the HyPhy package [71]. The best-fitting nucleotide model (GTR + G) was determined using the automated tool provided by Datamonkey. Five models were used: single likelihood ancestor counting (SLAC), fixed-effect likelihood (FEL), random effect likelihood (REL), fast unbiased bayesian approximation (FUBAR) and mixed effects model of evolution (MEME). SLAC is based on the reconstruction of the ancestral sequences and the counts of dS and dN at each codon position of the phylogeny. FEL estimates the ratio of dN/dS on a site-by-site basis, without assuming an a priori distribution across sites while REL fits a distribution of rates across sites and then infers the substitution rate for individual sites. FUBAR detects selection much faster than the other methods and to leverage Bayesian MCMC to robustly account for parameter estimation errors. Finally, MEME is capable of identifying instances of both episodic and pervasive positive selection at the level of an individual site. Sites with P values <0.1 for SLAC, FEL and MEME, posterior probability of >0.9 for FUBAR, and Bayes Factor >50 for REL were considered as being under selection. CODEML (PAML version 4, [72]) was also used to detect positive selection by comparing a null model and a model that allows positive selection (M1 vs. M2 and M7 vs. M8). The contrasting models were compared by computing twice the difference in the natural logs of the likelihoods (2ΔlnL). In the site-specific models that allow the ratio ω to vary among codons, we performed Likelihood Ratio Tests (LRTs) with 2 degrees of freedom to compare the following models (NS sites): M1 (nearly neutral evolution ω0 = 0, ω1 = 1) with M2 (neutral and positive selection: ω 0 = 0, ω 1 = 1, ω 2 > 1) and M7 (beta-distributed negative selection: 0 # ω # 1) with M8 (beta-distributed negative selection and positive selection: 0 # ω1 # 1, ω2 >1) [2,73]. Only amino acids identified in M8 by using the Bayes Empirical Bayes (BEB) approach and with posterior probability >95% were considered as evolving under positive selection. For the initial working topology, ML trees were constructed using MEGA5 [74] with substitution nucleotide models determined by the software: TN93 + I and partial deletion (95% cut-off) for PPP1R2P9 and K2 + G with G = 4 and partial deletion (95% cut-off) for PPP1R2.

### Sperm extracts and immunoprecipitation

Since testis is one of the organs where most pseudogenes are expressed [75] and spermatozoa are the final product of spermatogenesis, the presence of some of the studied pseudogenes was tested in human sperm. Ejaculated sperm was collected from healthy donors by masturbation into an appropriate sterile container. Spermograms were performed by experienced technicians and only samples with normal parameters were used [76]. Informed consents were signed allowing samples to be used for scientific purposes. The study was conducted in accordance with the guidelines of the “Helsinki Declaration”. In brief, sperm was lysed in 1 × RIPA buffer (radioimmunoprecipitation buffer, Millipore Iberica S.A.U., Madrid, Spain) supplemented with protease inhibitors (10 mM benzamidine, 1.5 μM aprotinin, 5 μM pepstatin A, 2 μM leupeptin, 1 mM PMSF), sonicated 3 × 10 sec and centrifuged at 16000 g for 20 min, at 4°C. RIPA supernatant sperm extract was immunoprecipitated using Dynabeads® Protein G (Life Technologies S.A., Madrid, Spain) and 1 μg of rabbit anti-PPP1R2 (against a mouse PPP1R2 peptide, amino acids 134–147) with standard direct immunoprecipitation procedure [48]. Also, an independent RIPA supernatant sperm extract was prepared, boiled in a water bath for 30 min, chilled on ice for 2 min and centrifuged at 16000 g for 20 min, 4°C to obtain a heat stable extract.

### Mass spectrometry

For mass spectrometry analysis, the immunoprecipitate and the heat stable extract were resolved by 10% SDS-PAGE along with purified positive controls. Gels were stained with Coomassie blue colloidal (Sigma-Aldrich Química, S.A., Sintra, Portugal) using standard procedures [48]. Bands were then excised from the gel using commercial PPP1R2 band as control and destained. An overnight digestion with trypsin (Promega, Madison, Wisconsin, USA) was performed and resulting peptides were extracted and prepared for mass spectrometry analysis using an Orbitrap Velos mass spectrometer as described elsewhere [48]. Subsequent generated data were imported to ProteinScapeTM (Bruker Daltonik GmbH, Bremen, Germany, [77]) and analyzed using MASCOT (version 2.2.0, Matrix Science, London, UK, [78]) search algorithm. Proteins were considered to be identified if the Mascot score (ProteinScapeTM) was higher than 65.

## Competing interests

The authors have declared that no competing interests exist.

## Authors’ contributions

Conceived and designed the bioinformatic studies: LKG, JA and PJE. Performed the bioinformatic studies: LKG, JA and JMF. Conceived and designed the experiments: LKG, TM and MF. Performed the experiments: LKG and TM. Analyzed the data: LKG, JA, JMF, TM and PJE. Contributed with reagents/materials/analysis tools: KM, OABCS, MF and PJE. Wrote the paper: LKG, JA, JMF, TM, MF and PJE. All authors read and approved the final manuscript.

## Supplementary Material

Additional file 1: Table S1 Nucleotide sequences used for the alignments and evolutionary analysis. Click here for file

Additional file 2: Table S2 PPP1R2 and PPP1R2P9 sites under negative and positive selection revealed by 5 different methods using the Datamonkey webserver. Click here for file
