# Supplementary material for: Not so pseudo: the evolutionary history of protein phosphatase 1 regulatory subunit 2 and related pseudogenes
Source: BMC Evol Biol. 2013 Nov 6;13:242. doi: 10.1186/1471-2148-13-242 (PMC3840573; doi:10.1186/1471-2148-13-242)
Supplement: Additional file 1: Table S1 — Nucleotide sequences used for the alignments and evolutionary analysis. [file 1471-2148-13-242-S1.docx]

Table S1. *Nucleotide sequences used for the alignments and evolutionary analysis.*

| **Order** | **Species** | **Gene Name** | **Alias** | **ReferenceID** | **Chr** | **ChrID** |
| --- | --- | --- | --- | --- | --- | --- |
| Primates | Human | PPP1R2 | I2 IPP2 | NM_006241.4 | 3q29 | NC_000003.11 |
|  | *Homo sapiens* | PPP1R2P1 |  | NG_027882.1 | 6p21.3 | NC_000006.11 |
|  |  | PPP1R2P2 |  | NG_000913.3 | 21q22.13 | NC_000021.8 |
|  |  | PPP1R2P3 | I2L | NR_038443.1 | 5q33.3 | NC_000005.9 |
|  |  | PPP1R2P4 |  | NG_011458.1 | 13q14.13 | NC_000013.10 |
|  |  | PPP1R2P10 |  | NG_029109.1 | 13q21.31 | NC_000013.10 |
|  |  | PPP1R2P5 |  | NG_021516.1 | 2q12.3 | NC_000002.11 |
|  |  | PPP1R2P6 |  | NG_022575.1 | 7q34 | NC_000007.13 |
|  |  | PPP1R2P8 |  | n.a. | 5p14.3 | NC_000005.9 |
|  |  | PPP1R2P9 | I4 IPP4 | NR_002191.2 | Xp11.3 | NC_000023.10 |
|  |  |  |  |  |  |  |
|  | Chimpanzee | PPP1R2 | I2 | XM_516963.3 | 3 | NC_006490.3 |
|  | *Pan troglodytes* | PPP1R2P1 |  | XR_127820.1 | 6 | NC_006473.3 |
|  |  | PPP1R2P2 |  | BS000199.1 | 21 | NC_006488.2 |
|  |  | PPP1R2P3 | I2L | XM_003310938.1 | 5 | NC_006472.3 |
|  |  | PPP1R2P10 |  | XM_003314161.1 | 13 | NC_006480.3 |
|  |  | PPP1R2P5 |  | XR_127157.1 | 2A | NC_006469.3 |
|  |  | PPP1R2P6 |  | AC144780.1 | 7 | NC_006474.3 |
|  |  | PPP1R2P8 |  | n.a. | 5 | NC_006472.3 |
|  |  |  |  |  |  |  |
|  | Orangutan | PPP1R2 | I2 IPP2 | XM_002814436.1 | 3 | NC_012594.1 |
|  | *Pongo abelii* | PPP1R2P1 |  | AC206576.3 | 6 | NW_002874546.1 |
|  |  | PPP1R2P2 |  | n.a. | 21 | NC_012612.1 |
|  |  | PPP1R2P3 | I2L | XM_002816121.1 | 5 | NC_012596.1 |
|  |  | PPP1R2P10 |  | XM_002824322.1 | 13 | NC_012604.1 |
|  |  | PPP1R2P10-Like |  | n.a. | 13 | NC_012604.1 |
|  |  | PPP1R2P5 |  | n.a. | 2A | NC_012592.1 |
|  |  | PPP1R2P6 |  | n.a. | 7 | NC_012598.1 |
|  |  | PPP1R2P9 | I4 IPP4 | XM_002831555.1 | X | NC_012614.1 |
|  |  |  |  |  |  |  |
|  | Gibbon | PPP1R2 | I2 IPP2 | XM_003280422.1 | scaf_234 (11) | NW_003501604.1 |
|  | *Nomascus leucogenys* | PPP1R2P1 |  | n.a. | scaf_81 (1b) | NW_003501451.1 |
|  |  | PPP1R2P2 |  | n.a. | scaf_32 (25) | NW_003501402.1 |
|  |  | PPP1R2P3 |  | n.a. | scaf_57 (2) | NW_003501427.1 |
|  |  | PPP1R2P5 |  | XM_003277451.1 | scaf_152 (14) | NW_003501522.1 |
|  |  | PPP1R2P6 |  | n.a. | scaf_71 (13) | NW_003501441.1 |
|  |  | PPP1R2P9 | I4 IPP4 | XM_003271019.1 | sca_72 (X) | NW_003501442.1 |
|  |  |  |  |  |  |  |
|  | Rhesus monkey | PPP1R2 | I2 IPP2 | XM_001097826.2 | 2 | NC_007859.1 |
|  | *Macaca mulatta* | PPP1R2P1 |  | n.a. | 4 | NC_007861.1 |
|  |  | PPP1R2P2 |  | n.a. | 3 | NC_007860.1 |
|  |  | PPP1R2P10 |  | n.a. | 17 | NC_007874.1 |
|  |  | PPP1R2P5 |  | AC187497 | 13 | NC_007870.1 |
|  |  | PPP1R2P9 | I4 IPP4 | XM_001088324.2 | X | NC_007878.1 |
|  |  |  |  |  |  |  |
|  | Grivet | PPP1R2P1 |  | AC241599.3 | n.a. | n.a. |
|  | *Chlorocebus aethiops* |  |  |  |  |  |
|  |  |  |  |  |  |  |
|  | Marmoset | PPP1R2 | I2 IPP2 | XM_002758211.1 | 15 | NC_013910.1 |
|  | *Callithrix jacchus* | PPP1R2P5 |  | n.a. | 14 | NC_013909.1 |
|  |  | PPP1R2P1 |  | AC242643.3 | 4 | NC_013899.1 |
|  |  | PPP1R2P6 |  | n.a. | 8 | NC_013903.1 |
|  |  | PPP1R2P9-Like |  | n.a. | X | NC_013918.1 |
|  |  | PPP1R2P9 | I4 IPP4 | XM_002762795.1 | X | NC_013918.1 |
|  |  |  |  |  |  |  |
| Artiodactyla | Pig | PPP1R2P9-Like | I4 IPP4 | XR_131237.1 | X | NC_010461.3 |
|  | *Sus scrofa* |  |  |  |  |  |
|  |  |  |  |  |  |  |
|  | Cow | PPP1R2 | I2 IPP2 | NM_001035392.1 | 1 | NC_007299.4 |
|  | *Bos taurus* | PPP1R2P9 | I4 IPP4 | NM_001079599.1 | X | NC_007331.3 |
|  |  |  |  |  |  |  |
|  | Horse | PPP1R2 | I2 IPP2 | XM_001500822.3 | 19 | NC_009162.2 |
|  | *Equus caballus* | PPP1R2P9 | I4 IPP4 | XM_001491737.1 | X | NC_009175.2 |
|  |  |  |  |  |  |  |
| Proboscidea | Elephant | PPP1R2P9-Like |  | XM_003418018.1 | n.a. | n.a. |
|  | *Loxodonta africana* |  |  |  |  |  |
|  |  |  |  |  |  |  |
| Carnivora | Giant panda | PPP1R2 | I2 IPP2 | XM_002921452.1 | scaf_1297 | NW_003217779.1 |
|  | *Ailuropoda melanoleuca* | PPP1R2-Like |  | n.a. | scaf_639 | NW_003217930.1 |
|  |  | PPP1R2P9 | I4 IPP4 | XM_002922902.1 | scaf_438 | NW_003217919.1 |
|  |  |  |  |  |  |  |
|  | Dog | PPP1R2 |  | XM_535781.2 | 33 | NC_006615.2 |
|  | *Canis lupus familiaris* | PPP1R2P9 |  | XM_548958.2 | X | NC_006621.2 |
|  |  |  |  |  |  |  |
| Lagomorpha | Rabbit | PPP1R2 |  | NM_001082744.2 | 14 | NC_013682.1 |
|  | *Oryctolagus cuniculus* | PPP1R2P9 |  | XM_002719841.1 | X | NC_013690.1 |
|  |  |  |  |  |  |  |
| Rodentia | Rat | PPP1R2 | I2-α1 I2-α2 | NM_138823.2 | 11 | NC_005110.2 |
|  | *Rattus norvegicus* | PPP1R2P9-Like | I2-β | XM_002730182.1 XM_002727659.1 | X | NC_005120.2 |
|  |  | PPP1R2P9 |  | XM_002730188.1 XM_002727510.1 NM_001106953.1 | X | NC_005120.2 |
|  |  |  |  |  |  |  |
|  | Mouse | PPP1R2 |  | NM_025800.3 | 16 | NC_000082.5 |
|  | *Mus musculus* | PPP1R2P9 |  | NR_033171.1 | X | NC_000086.6 |
|  |  | PPP1R2P9-Like |  | NR_033731.1 | X | NC_000086.6 |
|  |  |  |  |  |  |  |
| Marsupials | Opossum | PPP1R2-Like |  | XM_001371978.2 | n.a. | NW_001583292.1 |
|  | *Monodelphis domestica* |  |  |  |  |  |
|  |  |  |  |  |  |  |

*Nucleotide sequences were retrieved from GenBank and Ensembl databases.*
